# Variable Persister Gene Interactions with (p)ppGpp for Persister Formation in *Escherichia coli*

**DOI:** 10.3389/fmicb.2017.01795

**Published:** 2017-09-20

**Authors:** Shuang Liu, Nan Wu, Shanshan Zhang, Youhua Yuan, Wenhong Zhang, Ying Zhang

**Affiliations:** ^1^Key Lab of Molecular Virology, Institute of Medical Microbiology, Department of Infectious Diseases, Huashan Hospital, Fudan University Shanghai, China; ^2^Department of Molecular Microbiology and Immunology, Bloomberg School of Public Health, Johns Hopkins University, Baltimore MD, United States

**Keywords:** persistence, persister gene, ppGpp, knockout mutant, interactions

## Abstract

Persisters comprise a group of phenotypically heterogeneous metabolically quiescent bacteria with multidrug tolerance and contribute to the recalcitrance of chronic infections. Although recent work has shown that toxin-antitoxin (TA) system HipAB depends on stringent response effector (p)ppGppin persister formation, whether other persister pathways are also dependent on stringent response has not been explored. Here we examined the relationship of (p)ppGpp with 15 common persister genes (*dnaK*, *clpB*, *rpoS*, *pspF*, *tnaA*, *sucB*, *ssrA*, *smpB*, *recA*, *umuD*, *uvrA*, *hipA*, *mqsR*, *relE*, *dinJ*) using *Escherichia coli* as a model. By comparing the persister levels of wild type with their single gene knockout and double knockout mutants with *relA*, we divided their interactions into five types, namely A “dependent” (*dnaK*, *recA*), B “positive reinforcement” (*rpoS*, *pspF*, *ssrA*, *recA*), C “antagonistic” (*clpB*, *sucB*, *umuD*, *uvrA*, *hipA*, *mqsR*, *relE*, *dinJ*), D “epistasis” (*clpB*, *rpoS*, *tnaA*, *ssrA*, *smpB*, *hipA*), and E “irrelevant” (*dnaK*, *clpB*, *rpoS*, *tnaA*, *sucB*, *smpB*, *umuD*, *uvrA*, *hipA*, *mqsR*, *relE*, *dinJ*). We found that the persister gene interactions are intimately dependent on bacterial culture age, cell concentrations (diluted versus undiluted culture), and drug classifications, where the same gene may belong to different groups with varying antibiotics, culture age or cell concentrations. Together, this study represents the first attempt to systematically characterize the intricate relationships among the different mechanisms of persistence and as such provide new insights into the complexity of the persistence phenomenon at the level of persister gene network interactions.

## Introduction

Chronic and recalcitrant biofilm infections tolerant to antibacterial treatment pose a major medical problem, since they can cause considerable morbidity and frequently require multiple courses of antibiotic treatment, which in turn may contribute to the emergence of stable antibiotic resistance. Persisters are considered to play a predominant role in the recalcitrance of chronic bacterial infections by *Mycobacterium tuberculosis* and *Escherichia coli* (Zhang et al., 2012; Cui et al., 2016). They are well known for their survival in supra-lethal dose of multiple antibiotic and other environmental stresses. This phenomenon is widely present in virtually all microbes (Allison et al., 2011; Zhang et al., 2012; Feng et al., 2015; Xu et al., 2016). In contrast to antibiotic resistance, bacterial persister cells constitute a subpopulation of metabolically quiescent slow-growing or growth arrested cells with no heritable resistance mutations or increased minimal inhibitory concentration (MIC) compared to wild–type cells.

Although the exact mechanisms underlying persistence have yet to be uncovered, several pathways have been identified to be implicated in the formation of bacterial persisters (Zhang, 2014; Harms et al., 2016; Kaldalu et al., 2016; Van den Bergh et al., 2017). Since the discovery of *hipA*7 strain having a 100∼1000-fold increase in persister level (Moyed and Bertrand, 1983), at least 10 type II TA models have been reported to have an intimate association with persistence in *E. coli* (Lewis, 2010; Wang and Wood, 2011; Maisonneuve et al., 2013). However, the role of TA models in persisters is being challenged by some recent papers (Ramisetty et al., 2016; Van Melderen and Wood, 2017). In addition to TA genes, SOS response is required in persister formation under DNA damaging conditions, such as fluoroquinolones (Dorr et al., 2009). And indole also mediates persistence under nutrient-limiting conditions (Vega et al., 2012). Our previous studies have discovered energy production genes *sucB/ubiF*, *trans*-translation genes *ssrA/smpB*, and phosphate metabolism regulating gene *phoU*, being important for pesister formation (Li and Zhang, 2007; Ma et al., 2010; Li et al., 2013). (p)ppGpp, which is synthesized by RelA/SpoT from GDP or GTP (Hauryliuk et al., 2015) and rapidly accumulates during the stringent response (SR) under amino acid starvation, has been shown to be a critical metabolic mediator of persisters (Korch et al., 2003; Fung et al., 2010; Maisonneuve et al., 2013; Amato and Brynildsen, 2015; Germain et al., 2015). The SR orchestrates accommodation to various conditions (Steinchen and Bange, 2016) and its second messenger (p)ppGpp can lead to alteration of many cellular activities by downregulation of genes or enzymes for rapid growth and upregulation of genes for stress survival. Korch et al. (2003) first observed that TA module toxin *hipA*7 produced a high level of persistence which is dependent on (p)ppGpp synthesized by *relA* when exposed to antibiotics. Recently, it has been shown that not all type II TA modules involved in persistence require the activation of stringent response (Germain et al., 2015). However, it remains unclear whether other persister genes require (p)ppGpp to exhibit their persistence phenotype.

Although many persister genes have been identified so far, their impacts on persister levels differ considerably depending on different times and antibiotics being used (Wu et al., 2015). We have shown that different genes have different roles under the same antibiotic exposure (Wu et al., 2015). In addition, the same gene could exhibit varying importance to different antibiotics. The purpose of the present work is to investigate the interactions of the known persister genes with the (p)ppGpp pathway. By studying the differential phenotype of single gene knockout strains and double knockout strains with *relA*, we unraveled that different persister genes have distinct relationships with (p)ppGpp, which led to changing or even reversal in persister levels. Of the 15 persister genes (*dnaK*, *clpB*, *rpoS*, *pspF*, *tnaA*, *sucB*, *ssrA*, *smpB*, *recA*, *umuD*, *uvrA*, *hipA*, *mqsR*, *relE*, *dinJ*) we evaluated, only Δ*dnaK* and Δ*recA* (gentamicin and ampicillin) mutant strains were dependent on (p)ppGpp in terms of inhibiting persister formation while the other 13 persister genes fell in either synergistic, antagonistic, overtaking or irrelevant categories (see **Figures 6–8** and Supplementary Table S1). Our findings shed new light on the complex interactions of persister genes with the (p)ppGpp pathway.

## Materials and Methods

### Bacterial Strains and Growth Conditions

The strains used in this study were derived from wild type *E. coli* K12 strain W3110. Cells were routinely cultured in Luria-Bertani (LB) broth (10 g Bacto-tryptone, 5 g yeastextract, and 10 g NaCl/liter). Cells from -80°C stock were grown overnight to 10^9^ CFU/ml in LB in 17- by 100-mm polypropylene tubes, diluted 1:1000 into 4 mL LB broth, and grown to stationary phase (10^9^ CFU/ml) or log phase (10^8^ CFU/ml) at 37°C with shaking (200 rpm) unless otherwise stated.

### Knockout Mutant Construction

Deletion of persister genes made in the *E. coli* W3110 background was achieved by using the λ Red recombination system, as described by Datsenko and Wanner (2000). The deleted genes were stably replaced with a chloramphenicol resistance gene and this selectable marker was removed using pCP20 when needed. All mutants and plasmid insertions were confirmed by PCR and sequencing (Biosune). Further details of primers designed for this purpose and additional external primers used to verify the correct integration of the PCR fragments by homologous recombination are described in our previous work(Wu et al., 2015).

### Persister Assay

Persistence was measured by determining the bacterial survival as colony-forming units (CFUs) per 1mL after exposure to 200 μg/ml ampicillin, or 8 μg/ml norfloxacin, or 40 μg/ml gentamicin for stationary phase cultures undiluted as well as diluted (1:100) in some cases. Following overnight growth, 1 ml undiluted cultures or 1 ml diluted cultures (10 μl cultures and 990 μl LB) were transferred to a 1.5 ml Eppendorf tube and immediately treated with the above antibiotics and incubated at 37°C without shaking for different times. Stationary or log phase cultures (1 ml) without antibiotic exposure were included as controls in the persister assay. The initial cell number was checked by sampling 10 μl and serially diluting and plating on LB agar. The cell viability was measured by samples withdrawn at the desired time points, washed and serially diluted in PBS, followed by inoculation onto LB agar without antibiotics. The CFU counts were measured after overnight incubation at 37°C.

## Results

Since the well-known persister gene *hipA* was shown to depend on (p)ppGpp to mediate persistence, a growing number of persister studies have shifted their emphasis toward the role of (p)ppGpp and its correlation with other persister genes. However, it is still unclear to what extent other persister genes are dependent on (p)ppGpp. To address this question, we constructed double knockout mutants of 15 known persister genes (*dnaK*, *clpB*, *rpoS*, *pspF*, *tnaA*, *sucB*, *ssrA*, *smpB*, *recA*, *umuD*, *uvrA*, *hipA*, *mqsR*, *relE*, *dinJ*) with *relA* which encodes (p)ppGpp synthetase. Theoretically, if a single-gene knockout mutant that affects persister level is dependent on (p)ppGpp, then its double knockout mutant with *relA* will display a similar persister phenotype as the wild type will do. With this assumption, we determined the persister levels of the single and double knockout mutants as well as Δ*relA* mutant and the parent strain W3110. Early stationary phase cultures of the wild type and mutants were treated with ampicillin (200 μg/ml), norfloxacin (8 μg/ml) or gentamicin (40 μg/ml), and the persister levels were measured at different time points for each antibiotic. We found that among the 15 persister genes analyzed, only Δ*dnaK* and Δ*recA* were dependent on (p)ppGpp in terms of their effect on persister levels, while the other 13 persister genes (*clpB*, *rpoS*, *pspF*, *tnaA*, *sucB*, *ssrA*, *smpB*, *recA*, *umuD*, *uvrA*, *mqsR*, *relE*, *dinJ*) did not seem to be dependent on (p)ppGpp because their double knockout mutants exhibited different persister numbers compared with the parent strain W3110. However, in the case of *hipA* as a well-known example of ppGpp-dependent gene (Korch et al., 2003), the *hipA7* allele confers persistence in a manner that is dependent on ppGpp since lack of ppGpp due to *relA* mutation diminished the high persistence phenotype in the *hipA7*strain. However, in the case of *dnaK* and *recA* mutant cells, we found ppGpp is required for the opposite effect (i.e., lower persistence).

### DnaK and RecA Are Implicated in Persistence to Gentamicin and Ampicillin and Their Persistence Levels Are Dependent on (p)ppGpp

**FIGURE 1:**
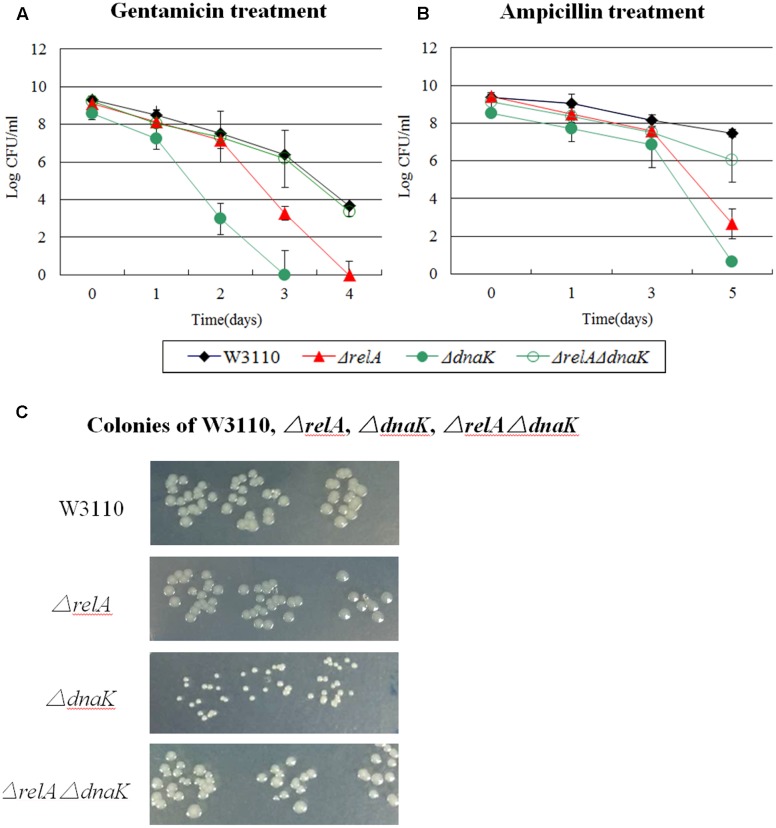
Effect of *dnaK* single and double-gene knockout mutations on *E. coli* persister formation. Cultures of W3110 and persister gene deletion mutants grown to stationary phase and immediately treated with **(A)** gentamicin (40 μg/ml) and **(B)** ampicillin (200 μg/ml). **(C)** Colonies of W3110, Δ*relA*, Δ*dnaK* and Δ*relA*Δ*dnaK* after 16 h incubation at 37°C on LB plates without antibiotics. Data shown are from three independent experiments with 3 replicates per experiment and error bars indicate the standard deviations.

We found that addition of three different antibiotics to early stationary phase culture of the Δ*dnaK* mutant indeed produced dramatically lower persister numbers compared with its parent strain W3110. The ratio was < 10^-6^ for gentamicin and ampicillin (see **Figures 1A,B**) and about 1:10 for norfloxacin, respectively. The Δ*relA* mutant also had a 10^3^∼10^4^ -fold lower (gentamicin and ampicillin) (see **Figures 1A,B**) and 10∼100-fold lower (norfloxacin) persister numbers than W3110. Interestingly, the Δ*relA*Δ*dnaK* mutant demonstrated a 10^3^∼10^6^ -fold higher persistence phenotype compared with either Δ*dnaK* or Δ*relA* and was similar to the level of the parent strain with gentamicin and ampicillin exposure when the cultures were taken from early stationary phase (see **Figures 1A,B**). However, with norfloxacin exposure, the persister numbers produced by Δ*relA*Δ*dnaK* remained the same as Δ*dnaK* when the cultures were taken from early stationary phase (5 h) (∼10 –fold decrease of either Δ*relA*Δ*dnaK* or Δ*dnaK* compared with W3110) (see **Figure 8**). At late stationary phase (18 h), the persister numbers of Δ*dnaK* kept at significant low levels for all the three antibiotics (10^6^∼10^8^ –fold lower for gentamicin and norfloxacin, ∼10^3^ –fold lower for ampicillin compared with W3110), but Δ*relA* showed a similar persister number as W3110 for all the antibiotics we tested. Although the persister levels of the Δ*relA*Δ*dnaK* mutant were the same as those of W3110 or Δ*relA* in the presence of gentamicin and ampicillin, and only < 10 –fold decrease compared with W3110 or Δ*relA* for norfloxacin, it was difficult to discriminate whether Δ*relA*Δ*dnaK* reverted back to W3110 or was the same as Δ*relA*. To address this, we chose the early stationary phase inocula for our subsequent test for all the 15 persister genes we tested.

It is important to note that the Δ*dnaK* mutant had a growth defect with characteristic small colonies on agar plates. However, this characteristic was diminished and reverted back to that of wild type strain W3110 in the case of Δ*relA*Δ*dnaK* (see **Figure 1C**). Together, the above results indicated the DnaK is an important factor for all the three antibiotic related persister formation pathways. The decreased persistence phenotype as well as its growth defect of Δ*dnaK* seemed to depend on functional (p)ppGpp. And this dependence had intimate relationship with the age of inocula and antibiotic classification. At early stationary phase, the Δ*relA*Δ*dnaK* mutant did not revert back to a phenotype similar to W3110 with norfloxacin exposure as it did with gentamicin and ampicillin. As for the age of inocula, we observed the reverted phenotype of Δ*relA*Δ*dnaK* only at early stationary phase, but with late stationary phase inocula, Δ*relA* and W3110 exhibited the same persister level. However, the assumption conflicted with the fact (p)ppGpp can trigger cells to enter persistent state. Because low (p)ppGpp concentration in Δ*relA* is supposed to accelerate the decrease of persisters in Δ*dnaK*, but instead Δ*relA* had the opposite effect in the Δ*dnaK* background.

The mutant strain of Δ*recA* which is involved in SOS response also exhibited the same phenomenon as Δ*dnaK* with gentamicin and ampicillin exposure in the early stationary phase. We observed that after 4 days of gentamicin treatment, the persister levels of Δ*relA* and Δ*recA* decreased below the limit of detection, but Δ*relA*Δ*recA* still had ∼10^2^ CFU/ml viable cells left although this number was lower (∼10^4^-fold decrease) than that of W3110 (∼10^6^) (see **Figure 6**). On the 5th day of ampicillin treatment, Δ*relA*Δ*recA* had almost 10^5^ CFU number while Δ*relA* and Δ*recA* had only 10^2^∼10^3^ CFU (see **Figure 7**).

The *dnaK* mutant was temperature sensitive and had a deficient growth even at 30°C. Therefore, to exclude the impact of impaired growth inherent in Δ*dnaK*, we analyzed the persister levels of W3110, Δ*relA*, Δ*dnaK* and Δ*relA*Δ*dnaK* at 25°C. As a result, the same phenomenon was observed for all the three antibiotics. That is, the persister level of Δ*relA*Δ*dnaK* was similar to that of the parent strain W3110 for gentamicin and ampicillin and was the same as Δ*dnaK* for norfloxacin (data not shown). Another evidence which supported that Δ*dnaK* did not obtain compensatory mutations was the small colonies during the period of persister analysis.

### (p)ppGpp or Other Persister Pathways Are Insufficient Alone and a Positive Reinforcement Is Necessary to Eliminate Persister Formation

**FIGURE 2:**
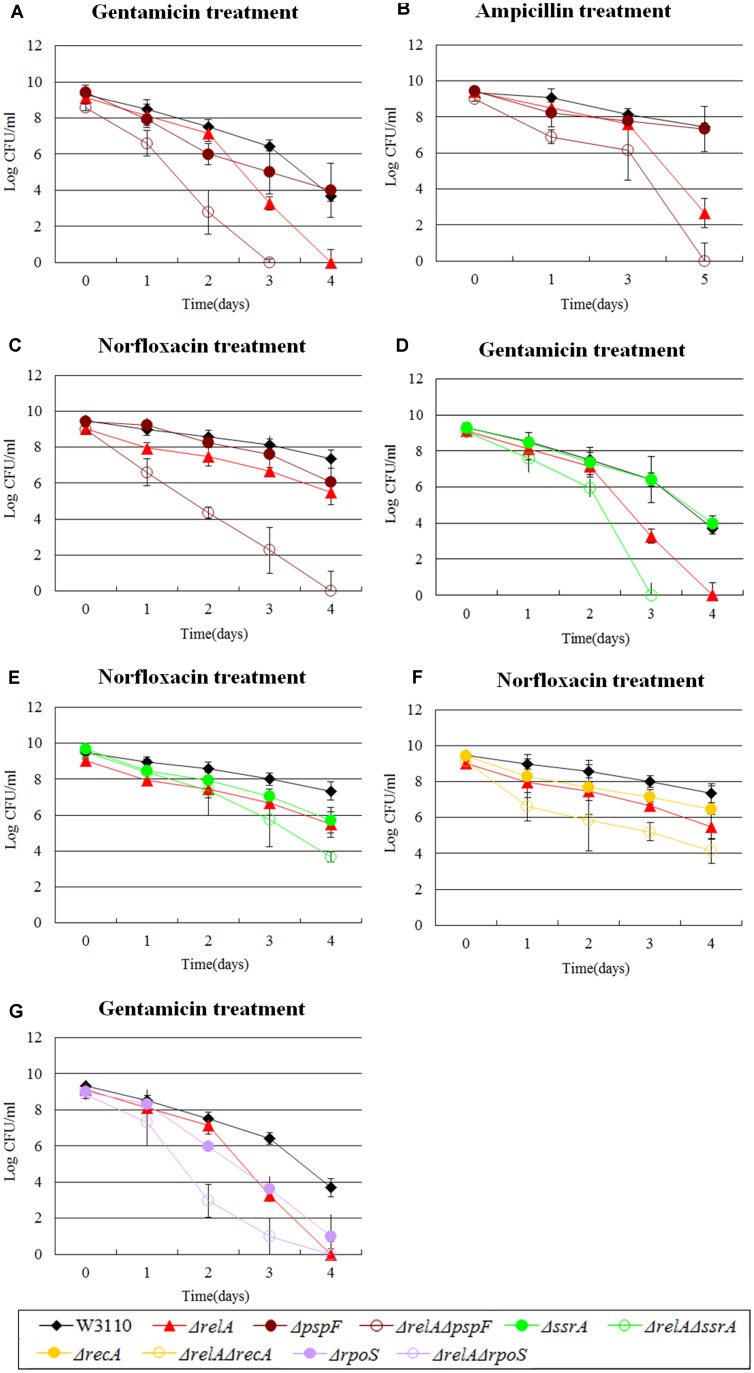
Effect of single-gene knockout mutations of *pspF, ssrA, recA* and *rpoS* and their double-gene knockout mutations with *relA* on *E. coli* persister formation. Early stationary phase cultures of W3110, Δ*relA* and **(A–C)** *pspF*, **(D,E)** *ssrA* knockout mutations, **(F)** *recA* and **(G)** *rpoS* knockout mutations were exposed to gentamicin (40 μg/ml), norfloxacin (8 μg/ml) and ampicillin (200 μg/ml) for 4–5 days at 37°C without shaking. Cells (10 or 200 μl) were removed, washed and plated to determine the persister numbers at the indicated time points. Data shown are from three independent experiments and error bars indicated the standard deviations.

Previously we have shown the hierarchy in importance of various persister genes (Wu et al., 2015). PspF is an enhancer-binding protein and a transcriptional activator of phage shock (Psp) system (Osadnik et al., 2015). While Psp pathway is involved in indole-induced persister formation (Vega et al., 2012), *pspF* was demonstrated to be less important than other persister genes (Wu et al., 2015). Here, we confirmed the results of our previous study, and found that the Δ*pspF* had a negligible impact on persister levels of the mutant for gentamicin and ampicillin exposure, and showed only a 10 fold defect in norfloxacin exposure. Unexpectedly, the double knockout mutant of *relA* and *pspF* significantly decreased the persister numbers (10^5^∼10^7^ -fold) compared with Δ*pspF* alone and was also more prominent than Δ*relA* (10^2^∼10^3^-fold decrease for gentamicin and ampicillin and >10^5^-fold reduction for norfloxacin) (see **Figures 2A–C**). In addition, we found that the colonies formed by Δ*relA*Δ*pspF* were smaller in size and grew more slowly than W3110 (the maximum CFU number of Δ*relAcccpspF* was only 10^8^). This is in contrast to *dnaK*, as the Δ*relA*Δ*pspF* double mutant caused considerable persister defect, and Δ*relA*Δ*pspF* mutant was rapidly killed by all the three antibiotics tested regardless of the stage of stationary phase.

Among the genes we tested, there were other three genes analogous to *pspF.* For example, *ssrA*, a trans-translation gene, mediates tolerance to multiple antibiotics (Li et al., 2013). Interestingly, Δ*relA*Δ*ssrA* exhibited a more significant deficiency in persister numbers when exposed to gentamicin and norfloxacin compared with W3110, and >10^6^-fold and >100-fold decrease were observed for Δ*relA* or Δ*ssrA* in gentamicin and norfloxacin treatment, respectively (see **Figures 2D,E**). Similar behavior was observed for *recA* (SOS response) and *rpoS* (global regulator). Their double knockout mutants with *relA* were more susceptible to just one antibiotic (norfloxacin or gentamicin), with 10∼100-fold decrease for Δ*relA*Δ*recA* and >1000-fold decrease for Δ*relA*Δ*rpoS* (at day 2∼3) compared to W3110 and their respective single gene deletion mutants (see **Figures 2F,G**). These findings demonstrated that a synergistic effect existing between these genes (*pspF*, *ssrA*, *recA*, and *rpoS*) and (p)ppGpp in undermining the formation of persisters, because neither Δ*relA* nor the single gene knockout mutants of the four genes could produce such significant persister deficiency. Although our data shown above indicated the persister level of Δ*recA* might be dependent on (p)ppGpp when challenged with gentamicin and ampicillin, it is not the case in the presence of norfloxacin. The phenomenon once again confirmed our previous study that the persistence phenomenon is not fixed but is in a dynamic state (Zhang, 2014; Wu et al., 2015).

### An Antagonistic Effect between (p)ppGpp and Some Persister Genes

**FIGURE 3:**
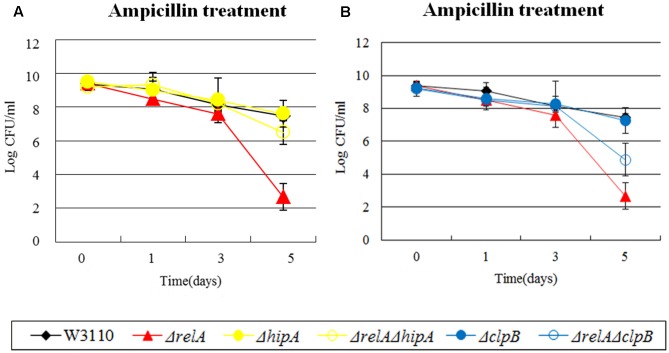
Effect of single-gene knockout mutations of *hipA* and *clpB* and their double-gene knockout mutations with *relA* on *E. coli* persister formation under ampicillin treatment. Early stationary phase cultures of W3110, Δ*relA* and **(A)** *hipA* or **(B)** *clpB* knockout mutations were exposed to ampicillin (200 μg/ml) for 4–5 days at 37°C without shaking. Cells (10 or 200 μl) were removed, washed and plated to determine the persister number at the indicated time points. Data shown are from three independent experiments and error bars indicate the standard deviations.

In our study, the persister levels of 8 double knockout mutants (Δ*relA*Δ*clpB*, Δ*relA*Δ*hipA*, Δ*relA*Δ*mqsR*, Δ*relA*Δ*relE*, Δ*relA*Δ*dinJ*, Δ*relA*Δ*umuD*, Δ*relA*Δ*uvrA*, Δ*relA*Δ*sucB*) fell between those of Δ*relA* and their own single gene deletion mutants. Based on the previous research, the high persistence of *hipA*7 mutant was eliminated when *relA* was deleted during treatment of penicillin. Therefore, we explored whether Δ*hipA* had the same connection with Δ*relA* as *hipA*7 did. Data shown here indeed supported our assumption in the treatment of ampicillin, where Δ*relA*Δ*hipA* decreased the persister number (∼10 -fold change) compared with W3110 and Δ*hipA* but still higher than that of Δ*relA* (∼10^4^-fold higher) (see **Figure 3A**). However, Δ*hipA* did not exibit an obvious change in persister numbers compared to W3110 when challenged to ampicillin. The results were not unexpected, because previous work in our lab have found the impact of Δ*hipA* on persisters was not obvious (Wu et al., 2015). We attributed this distinction to different assay methods and conditions. Another heat shock protein *clpB*, also exhibited the similar phenomenon where the persister level of Δ*relA*Δ*clpB* was between those of Δ*relA* and Δ*clpB* in the presence of ampicillin (see **Figure 3B**).

### The Impact of (p)ppGpp on Persistence Far Outweighs That of Some Persister Genes

**FIGURE 4:**
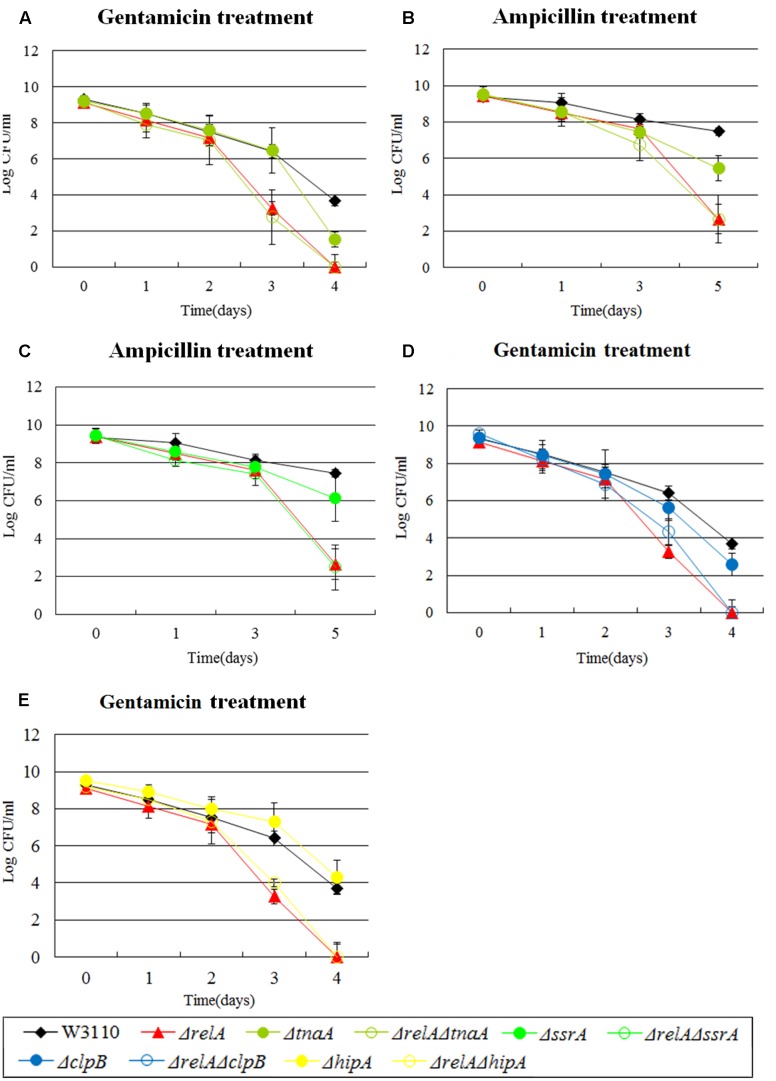
Effect of single-gene knockout mutations of *tnaA*, *ssrA*, *clpB* and *hipA* and their double-gene knockout mutations with *relA* on *E. coli* persister formation under gentamicin or ampicillin treatment. Early stationary phase cultures of W3110, Δ*relA* and **(A**,**B)** *tnaA*, **(C)** *ssrA*, **(D)** *clpB* and **(E)** *hipA* knockout mutations were exposed to gentamicin (40 μg/ml) or ampicillin (200 μg/ml) for 4–5 days at 37°C without shaking. Cells (10 or 200 μl) were removed, washed and plated to determine the persister number at the indicated time points. Data shown are from three independent experiments and error bars indicate the standard deviations.

For some genes, we discovered that the impact of their double knockout mutants on persistence was all but similar to Δ*relA* when exposed to certain antibiotics. For example, in the presence of gentamicin and ampicillin, a ∼10^2^-fold lower persister number was observed for Δ*tnaA* than W3110 while Δ*relA*Δ*tnaA* had a defect which was the same as Δ*relA* (10^4^∼10^5^-fold decrease compared with W3110) (see **Figures 4A,B**). Likewise, the extent of decrease in persister levels of Δ*relA*Δ*ssrA*, Δ*relA*Δ*clpB* and Δ*relA*Δ*hipA* was almost identical to Δ*relA* on exposure to ampicillin and (or) gentamicin (see **Figures 4C–E**). *rpoS* and *smpB* were also in this group when challenged with norfloxacin or ampicillin.

### The Persister Levels of Some Persister Gene Mutants Are Independent of (p)ppGpp

In our study, we found persister levels of 12 persister genes (*dnaK*, *clpB*, *rpoS*, *tnaA*, *sucB*, *smpB*, *umuD*, *uvrA*, *hipA*, *mqsR*, *relE*, *dinJ*) were not affected by (p)ppGpp in the presence of at least one antibiotic. That is, the double knockout mutants showed a similar persister level to their single-gene knockout mutants (see Supplementary Table S1 and **Figures 6**–**8**). Although we considered that the low persister levels of Δ*dnaK* in gentamicin and ampicillin was dependent on ppGpp as Δ*relA*Δ*dnaK* had a similar persister phenotype to W3110, it seemed *dnaK* was independent of (p)ppGpp in the treatment of norfloxacin for the similar persister numbers of Δ*dnaK* and Δ*relA*Δ*dnaK*. For *clpB, rpoS*, *tnaA, sucB, smpB*, *umuD*, *uvrA*, *hipA*, *mqsR*, *relE* and *dinJ*, they also occupied at least two categories (A, B, C, D, or E). This indicated that they could not only be “positive reinforcement”/“antagonistic”/“epistasis” with (p)ppGpp but also can be independent of (p)ppGpp in the presence of some specific antibiotics.

### Stationary Phase Culture Are More Suitable for Analyzing the Interaction of Persister Genes than Log Phase Cultures

**FIGURE 5:**
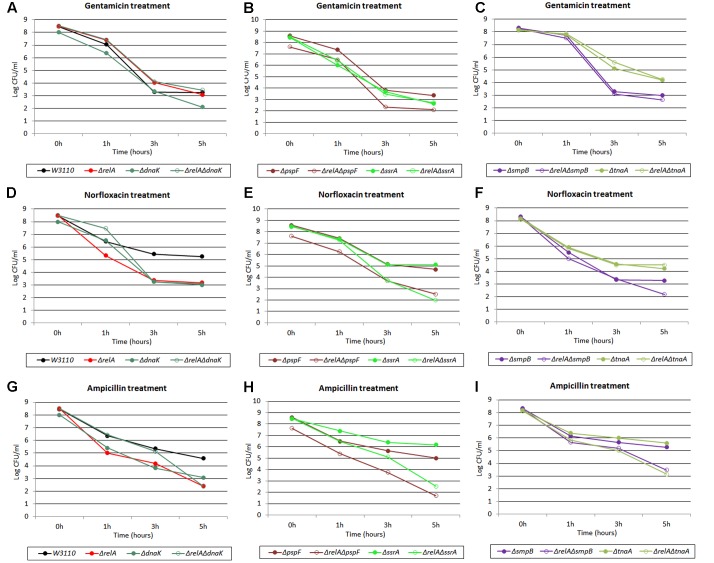
Killing curves of W3110, Δ*relA*, Δ*dnaK*, Δ*relA*Δ*dnaK*, Δ*pspF*, Δ*relA*Δ*pspF*, Δ*ssrA*, Δ*relA*Δ*ssrA*, Δ*tnaA*, Δ*relA*Δ*tnaA*, Δ*smpB* and Δ*relA*Δ*smpB* under three antibiotics treatment in log phase stage. Log phase cultures of the 10 strains were exposed to **(A–C)** gentamicin (30 μg/ml), **(D–F)** norfloxacin (8 μg/ml) and **(G–I)** ampicillin (150 μg/ml) for 5 h at 37°C without shaking. Cells (10 or 200 μl) were removed, washed and plated to determine the persister number at the indicated time points.

Diverse methods have been applied for persister measurement by many research groups (e.g., diluted or undiluted cultures, log phase or stationary phase cultures, shaking or without shaking and CFU or OD adjusted) (Orman and Brynildsen, 2015; Volzing and Brynildsen, 2015; Wu et al., 2015; Chowdhury et al., 2016; Shan et al., 2017). However, each method had its limitations/shortcomings. For example, in the case of diluted cultures, bacteria would become so sensitive to antibiotics that the persister levels often failed to be detected. The problem with log phase culture is the discordant growth rates of different mutants which could result in different initial CFU for different mutant strains. Here, to avoid the above potential issues, we chose stationary phase cultures without dilution to enrich persisters to determine the persister levels in our mutant strains. Although biphasic killing curves are often used to demonstrate the persister phenomenon, they were not seen for most strains (see **Figures 1**–**4**). To ensure the appropriateness of our methods utilizing stationary phase cultures, we randomly selected 5 pairs of single and double gene knockout mutants and subjected them to killing curve analysis along with W3110 and Δ*relA* using exponential phase cultures typically used in biphasic killing curve as demonstration of persister phenomenon. To exclude the changes in bacterial density, the survival curves of these strains were performed in the absence of antibiotics simultaneously. As expected, no significant decrease in cell viability were observed for the 10 mutant strains (data not shown). From **Figures 5A–F**, we observed in the treatment of gentamicin or norfloxacin, only few displayed atypical biphasic hallmark (Δ*relA*Δ*smpB*, Δ*relA*Δ*pspF*, Δ*relA*Δ*ssrA* in norfloxacin treatment and Δ*dnaK* in gentamicin treatment), while the majority of strains showed typical biphasic killing curves. However, in the presence of ampicillin treatment, the killing curves of six of the mutant strains did not show the biphasic characteristic (Δ*relA*, Δ*relA*Δ*dnaK*, Δ*relA*Δ*smpB*, Δ*relA*Δ*pspF*, Δ*relA*Δ*ssrA*, Δ*relA*Δ*tnaA*) (see **Figures 5G–I**). In addition, the interaction categories in log phase were also different from those in stationary phase (see **Table 1**). For example, when withdrawn from log phase, *ssrA* and *tnaA* fell in type E while they were categorized as type B or D in stationary phase in gentamicin treatment. In the presence of ampicillin, instead of depending on (p)ppGpp, *dnaK* was classified as “epistasis” group when cultures were withdrawn from log phase. Categories of *tnaA* and *smpB* also underwent changes from type D in stationary phase to type C in log phase. When exposed to norfloxacin, category changes were only seen in *tnaA* (type C in log phase and type E in stationary phase).

**Table 1:** The effect of the interaction of persister genes *pspF, ssrA, dnaK, smpB*, and *tnaA* with (p)ppGpp varies in different cultures in the presence of three cidal antibiotics.

Antibiotics	Gene	Log	Stationary	1:100 diluted
treatment	name	phase	phase	stationary cultures
	*pspF*	Type B	Type B	Type E
	*ssrA*	Type E	Type B	Type B
Gentamicin	*dnaK*	Type A or D^a^	Type A	Type A
	*smpB*	Type E	Type E	Type B
	*tnaA*	Type E	Type D	Type E
	*pspF*	Type B	Type B	Type B
	*ssrA*	Type B	Type B	Type E
Norfloxacin	*dnaK*	Type D or E^b^	Type E	Type D
	*smpB*	Type D or E	Type E	Type E
	*tnaA*	Type C	Type E	Type E
	*pspF*	Type B	Type B	Type B
	*ssrA*	Type D	Type D	Type E
Ampicillin	*dnaK*	Type D	Type A	Type D
	*smpB*	Type C	Type D	Type D
	*tnaA*	Type C	Type D	Type D

^a^*Type A or D means the persister level of Δ*relA* was similar to that of W3110 and it was hard to determine which type the *dnaK* belonged to when log phase cultures were exposed to gentamicin.^b^Type D or E means the persister level of the genes was similar to that of Δ*relA*, so it was difficult to determine which type the persister genes belonged to when log phase cultures were exposed to norfloxacin*.

### Using Undiluted Cultures for Persister Assay Is More Beneficial for Identifying Genes that Interact with (p)ppGpp

Persister levels of *E. coli* parent strain W3110 and 11 randomly selected mutant strains (*dnaK*, *clpB, pspF, tnaA, sucB, ssrA*, *smpB, recA, hipA, mqsR, relE*) were also measured using (1:100) diluted cultures as described in “Materials and Methods.” A comparison of the results obtained from the two assay methods (undiluted and diluted) revealed that the interaction categories were also intimately related to experimental methods (see **Table 1** and Supplementary Tables S1, S2). For example, instead of being dependent on (p)ppGpp under gentamicin and ampicillin treatment, *recA* was independent of (p)ppGpp for ampicillin (type E) and had a reinforcement effect with (p)ppGpp for gentamicin (type B). *pspF*, which belongs to type B (positive reinforcement) under all the three antibiotics when tested using undiluted cultures, was independent of (p)ppGpp for gentamicin when diluted cultures were used. Other genes, such as *tnaA* and *sucB*, also turned out to be independent of (p)ppGpp under gentamicin treatment. Taken together, our results indicated when cultures were diluted, the relationships (type A/B/C/D) of some genes with (p)ppGpp tended to become type E (irrelevant). This suggests that more genes dependent on (p)ppGpp would be revealed when undiluted cultures are used.

## Discussion

Bacteria often reside in environments with myriad stresses that require the immediate switching to proper physiological state in response to new conditions. Previous studies have revealed multiple genes belonging to different pathways being involved in the formation of persisters (Zhang, 2014), however, they mainly focus on one single gene or one pathway at a time and their epistatic interactions and relationships in the context of persister gene/pathway network are mostly unknown. Our findings presented here provide new insights about the mechanisms of persisters. Although previous studies have carried out research on the role of (p)ppGpp in persistence, the majority of them involved starvation stress or only one antibiotic. For example, Korch and colleagues showed *hipA*7 mutant had increased persister cells dependent on (p)ppGpp synthesis using one antibiotic (penicillin) (Korch et al., 2003). It has also been shown that loss of trans-translation genes (*ssrA*/*smpB*) and *clp* system decreased persister formation through (p)ppGpp in ampicillin treatment and diauxie (Amato and Brynildsen, 2015). Here we systematically addressed the interactions of different persister genes with the (p)ppGpp pathway using three classical bactericidal antibiotics (gentamicin, ampicillin and norfloxacin) in persister assays using stationary phase cultures. Unexpectedly, most of the 15 common persister genes we tested had relationships with (p)ppGpp to at least one of the antibiotics for early stationary phase (5 h) cultures and their connections varied according to the antibiotics. Furthermore, we found the connections of (p)ppGpp with some genes in certain antibiotic exposures completely disappeared when the inocula were taken from stationary phase (18 h). Our findings suggest that the interactions between the common persister genes and (p)ppGpp are drug-specific and culture age-dependent.

**FIGURE 6:**
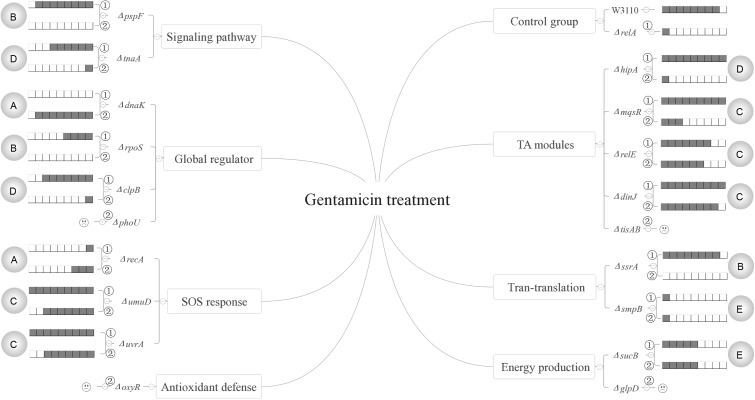
Models of the five relationships of (p)ppGpp and 15 persister genes in the presence of gentamicin. The gray circles on both flanks of the figures represent the 5 classifications of the 15 persister genes, A “dependent,” B “positive reinforcement,” C “antagonistic,” D “epistasis” and E “irrelevant”; The relative persister levels of W3110, Δ*relA* and single or double-gene knockout mutant strains of the 15 persister genes are presented as the gray part of the columns; 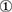 and 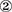 refer to single and double-gene knockout mutant strains, respectively. The “
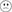
” symbolized the double-gene knockout mutant strains of four persister genes (*phoU*, *oxyR*, *tisAB*, *glpD*) failed to be constructed.

**FIGURE 7:**
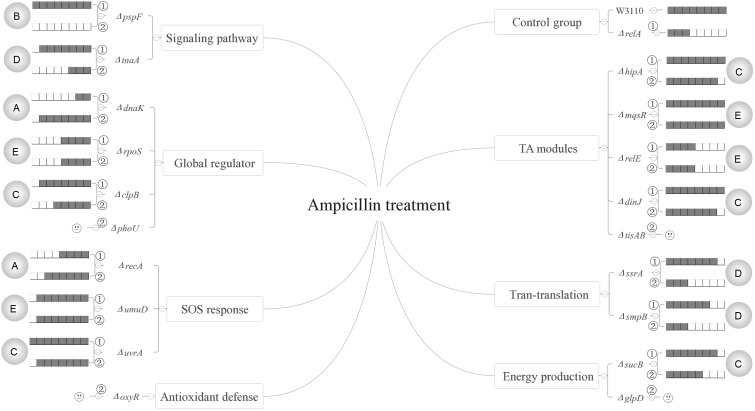
Models of the five relationships of (p)ppGpp and 15 persister genes in the presence of ampicillin. The gray circles on both flanks of the figures represent the five classifications of the 15 persister genes, A “dependent,” B “positive reinforcement,” C “antagonistic,” D “epistasis” and E “irrelevant”; The relative persister levels of W3110, Δ*relA* and single or double-gene knockout mutant strains of the 15 persister genes are presented as the gray part of the columns; 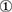 and 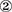 refer to single and double-gene knockout mutant strains, respectively. The “
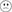
” symbolized the double-gene knockout mutant strains of four persister genes (*phoU*, *oxyR*, *tisAB*, *glpD*) failed to be constructed.

**FIGURE 8:**
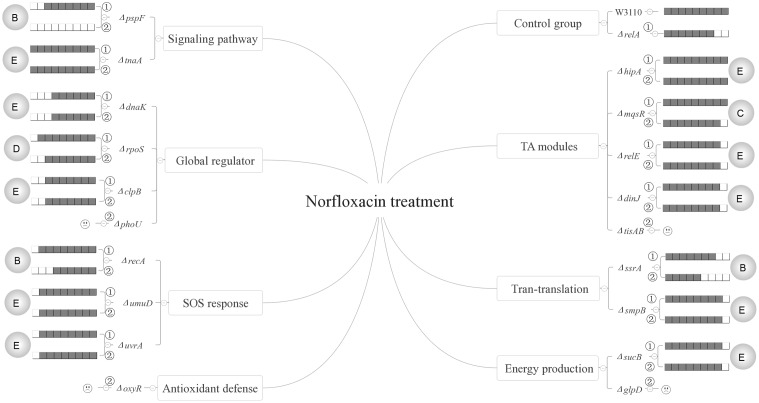
Models of the five relationships of ppGpp and 15 persister genes in the presence of norfloxacin. The gray circles on both flanks of the figures represent the five classifications of the 15 persister genes, A “dependent,” B “positive reinforcement,” C “antagonistic,” D “epistasis” and E “irrelevant”; The relative persister levels of W3110, Δ*relA* and single or double-gene knockout mutant strains of the 15 persister genes are presented as the gray part of the columns; 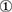 and 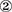 refer to single and double-gene knockout mutant strains, respectively. The “
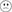
” symbolized the double-gene knockout mutant strains of four persister genes (*phoU*, *oxyR*, *tisAB*, *glpD*) failed to be constructed.

Based on our results, the persister genes we tested have complex interactions with ppGpp and could be divided into five categories for all the three antibiotics according to their relationships with (p)ppGpp (see **Figures 6**–**8**). The first group includes genes that affect persister level in a (p)ppGpp dependent manner. Among the genes (*dnaK*, *clpB*, *rpoS*, *pspF*, *tnaA*, *sucB*, *ssrA*, *smpB*, *recA*, *umuD*, *uvrA*, *hipA*, *mqsR*, *relE*, *dinJ*) we tested, only *dnaK* (global regulator) and *recA* (SOS response) belong to this group. Although the *dnaK* mutant was shown to have (p)ppGpp accumulation at high temperatures (Brown et al., 2002), it was not tested under antibiotic conditions for persistence phenotype. Therefore, we hypothesized there might be also an increasing concentration of (p)ppGpp in Δ*dnaK* under antibiotics treatment. If it is true, the double knockout mutant with *relA* will exhibit a lower pesister level than Δ*dnaK* did. However, contrary to our assumption, instead of reducing persister level of Δ*dnaK* further, Δ*relA*Δ*dnaK* exhibited a higher persister level than both Δ*dnaK* and Δ*relA* as it even reverted to the level of wild type strain W3110. It seems that the persister defect of Δ*dnaK* depended on a high concentration of (p)ppGpp. But this assumption contradicts with the well-known fact that high (p)ppGpp levels induce persister formation and drug tolerance. Another possibility is that there may be other pathways to mediate the regulation of Δ*relA*Δ*dnaK*. These pathways may be activated only when *dnaK* expression and (p)ppGpp are both at low levels, and their role is to maintain survival of bacteria. Therefore, further investigation of the mechanism is needed in future studies.

The second category is that persister genes have positive reinforcement effect with (p)ppGpp in persister formation. We defined it as “positive reinforcement effect” because their double knockout mutants had lower persister levels than either their own single gene mutants or Δ*relA*. Genes in this group are involved in pathways of trans-translation (*ssrA*), SOS response (*recA*), a global regulator (*rpoS*), and indole signaling pathways (*pspF*). Of these genes, *pspF* showed such a relationship with (p)ppGpp for all the three antibiotics while *ssrA* did so for gentamicin and norfloxacin. For *recA* and *rpoS*, we observed this effect only in the presence of norfloxacin or gentamicin. The results presented here may have two explanations. The first is that the two pathways affect persister formation independently and consequently lead to the lower persister levels. But this explanation does not seem to apply for *pspF* in ampicillin treatment and *ssrA* in gentamicin treatment. Because Δ*pspF* and Δ*ssrA* produced similar persister levels as W3110, the persister levels of their double-gene knockout mutants with *relA* were expected to be the same as Δ*relA* accordingly, but surprisingly the two double knockout mutants exhibited much lower persister levels than Δ*relA* did. Because Psp pathway is involved in indole-induced persister formation (Vega et al., 2012), and this suggests that there may be an interaction between stringent response and indole signaling pathways. We propose that there may be an induction of (p)ppGpp in Δ*pspF* which maintains its persister level. When we delete *relA* in Δ*pspF*, the (p)ppGpp induction is impaired which results in the significantly low persister level. Or another explanation is that the higher persister levels in single-gene knockout mutants (Δ*pspF*, Δ*ssrA*, Δ*recA* and Δ*rpoS*) compared with their double-gene knockout strains required the production of (p)ppGpp, so when *relA* was deleted, (p)ppGpp production sharply decreased and resulted in the low persister levels of the double knockout mutants. And it is readily adaptable for *rpoS*. Previous work showed the induction of (p)ppGpp by IPTG may positively regulate RpoS in MG1655 via *dksA* (Brown et al., 2002). Besides, (p)ppGpp is a plausible participant in stresses associated with RpoS regulation (Lange and Hengge-Aronis, 1994; Cassels et al., 1995; Hengge-Aronis, 1996; Loewen et al., 1998). Kayama and colleagues also revealed that in *Pseudomonas aeruginosa*, *rpoS* implemented its role in ofloxacin tolerance through (p)ppGpp although the exact mechanism involved in ofloxacin tolerance was not elucidated (Kayama et al., 2009). Based on the above reasons, we proposed that in terms of persister formation of *E. coli*, global regulators RpoS and (p)ppGpp are also interrelated and interact with each other, they may be both induced to maintain bacterial survival when exposed to antibiotic (gentamicin). When either of the two genes is deleted, the surviving cells will decrease. However, the extent of decrease is less than that when they are both deleted. As for single-gene deletion mutant, another gene can still function in other pathways involved in persistence. It is also possible that the four genes (*pspF*, *ssrA*, *recA* and *rpoS*) may play a key role in maintaining the low concentration of (p)ppGpp in Δ*relA* via SpoT, another (p)ppGpp synthetase with a weak synthetic activity. Deletion of them can severely affect the production of ppGpp, and consequently lead to the lower persister levels. This will be tested in future studies.

Genes in the third category have a common feature where their persister levels of double knockout mutants lie between the single gene deletion mutants and Δ*relA*. So genes in this group are termed “antagonistic” with (p)ppGpp. Pathways involved in this category are SOS response (*umuD/uvrA*), energy production (*sucB*), TA model (*hipA/mqsR/relE/dinJ*) and heat shock protein (*clpB*). Of the 8 double knockout mutants (Δ*relA*Δ*umuD*, Δ*relA*Δ*uvrA*, Δ*relA*Δ*sucB*, Δ*relA*Δ*hipA*, Δ*relA*Δ*mqsR*, Δ*relA*Δ*relE*, Δ*relA*Δ*dinJ*, Δ*relA*Δ*clpB*), they all exhibited lower persister levels than their own single gene knockout strains. It is worthwhile to note that although previous studies have reported ectopic expression of *hipA* can lead to increased persister numbers by inducing (p)ppGpp production via RelA in the treatment of β-lactam or quinolone antibiotic(Korch et al., 2003; Bokinsky et al., 2013; Germain et al., 2013). However, all the research focused on the induction of (p)ppGpp by HipA overexpression. Here we attempted to explore if (p)ppGpp is related with *hipA* of W3110 by observing the persister levels in Δ*hipA* and Δ*relA*Δ*hipA*. The fact that Δ*hipA* did not exhibit obvious low persister levels relative to W3110 in the treatment of ampicillin is consistent with our previous work. However, there was discrepancy between our present and previous work (Wu et al., 2015) in the presense of gentamicin (Δ*hipA* exhibited a little higher persister level than W3110 in this study). The assay methods and experimental conditions may be the major contributing factors because cultures we used in this study were not diluted as our previous research did. Besides, additional deletion of *relA* in Δ*hipA* had a lower persister level compared with Δ*hipA* and W3110. This phenomenon indicates that HipA may have an antagonistic effect with (p)ppGpp in persister formation under ampicillin treatment. Further exploration is required to elucidate whether other genes in this group would impact the concentration of (p)ppGpp.

The fourth group of *rpoS* (norfloxacin), *clpB* (gentamicin), *ssrA/smpB* (ampicillin), *tnaA* (gentamicin and ampicillin) and *hipA* (gentamicin) are supposed to be controlled by or dependent on (p)ppGpp or (p)ppGpp regulated genes. The last group contains most of the genes (*dnaK*, *clpB*, *rpoS*, *tnaA*, *sucB*, *smpB*, *mqsR*, *umuD*, *uvrA*, *hipA*, *relE*, *dinJ*). Genes in this category may be irrelevant to (p)ppGpp because additional deletion of *relA* did not bring about different persister levels compared with their single-gene mutants.

Notably, based on our data, only two genes displayed (p)ppGpp-dependent behavior while most persister genes we tested did not depend on (p)ppGpp in our persister assay. These results indicate that (p)ppGpp might not be so important, which challenges the current thinking (Amato et al., 2013; Amato and Brynildsen, 2015). Future studies with more persister genes and stresses should be performed to validate this finding.

Furthermore, by analyzing the killing curves of 10 selected mutants as well as W3110 and Δ*relA* in both log phase and stationary phase cultures, we found that hallmark of persistence as commonly shown with biphasic killing curve may not be applicable to cultures from stationary phase when challenged with the three antibiotics without culture dilution. Besides, while the majority of log phase cultures of the mutants displayed the characteristic biphasic killing in the presence of gentamicin and norfloxacin, 50% of the 12 persister gene mutant strains we tested did not show typical biphasic killing kinetics when challenged with ampicillin. Thus, it would appear that the biphasic killing hallmark of persistence is related to the bacterial growth stage and antibiotics, which means cultures withdrawn from stationary phase or cultures treated with ampicillin would not have biphasic killing curves. We therefore emphasize that there is limitation of solely relying on biphasic killing as the standard for persister demonstration, as is commonly believed or practiced in the field. This non-biphasic killing curves in persister assay are also commonly used by other research groups. For example, when Iris Keren analyzed the persister levels of *M. tuberculosis* under different concentrations of streptomycin, ciprofloxacin or rifampin, most killing curves did not display biphasic characteristic (Keren et al., 2011). Had we relied on the biphasic killing as the sole criterion for persister demonstration using log phase, we would have missed detection of significant relationships of persister genes with (p)ppGpp that are only observed in stationary phase instead of log phase used for biphasic killing demonstration. Besides, a comparison of two different persister assay methods (undiluted or diluted) in our study revealed that in order to exploit more (p)ppGpp-dependent genes, undiluted cultures may be a better choice. In addition, while previous studies indicate that culture age, inoculum size, type of antibiotics all affect persister levels for single persister genes (Li and Zhang, 2007; Luidalepp et al., 2011), an important observation of the current study is that these factors also affect the persister gene interactions and their types, suggesting a variable and plastic or adaptive persister gene interaction network in response to these changes. Future studies on more genes in different persister pathways are needed to gain a more comprehensive understanding of the persister gene interaction network. Such improved understanding will be important for developing more effective drugs killing persisters for improved treatment of persistent bacterial infections.

## Author Contributions

YZ, WZ, and SL conceived and designed the experiments. SL, NW, SZ, and YY performed the gene knockout experiments. SL performed the data analysis. SL wrote the manuscript.

## Conflict of Interest Statement

The authors declare that the research was conducted in the absence of any commercial or financial relationships that could be construed as a potential conflict of interest.
