# Supplementary material for: Variable Persister Gene Interactions with (p)ppGpp for Persister Formation in Escherichia coli
Source: Front Microbiol. 2017 Sep 20;8:1795. doi: 10.3389/fmicb.2017.01795 (PMC5611423; doi:10.3389/fmicb.2017.01795)
Supplement: Supplementary file 1 [file Table_1.DOCX]

Supplementary Material

Variable Persister Gene Interactions with (p)ppGpp for Persister Formation in *Escherichia coli*

Shuang Liu^1^, Nan Wu^1^, Shanshan Zhang^1^, Youhua Yuan^1^, Wenhong Zhang^1^,^*^ and

Ying Zhang^1,2,*^

*** Correspondence:**Ying Zhang
yzhang@jhsph.edu

WenHong Zhang
[zhangwenhong@fudan.edu.cn](mailto:zhangwenhong@fudan.edu.cn)

# Table S1 | Complex interactions of persister genes with stringent response (p)ppGpp in the presence of three cidal antibiotics^a^.

| **Name** | **Double knockout mutants with *relA*** | **Persister level comparison** | **Classification** | **Persister pathways** |
| --- | --- | --- | --- | --- |
| *dnaK* | *△relA△dnaK* | S^b^ < D^c^ (GEN/AMP) S = D^d^ (NOR) | A (GEN/AMP) E (NOR) | Global regulator |
| *clpB* | *△relA△clpB* | S > D (GEN/AMP) S = D (NOR) | C (AMP) D (GEN) E (NOR) | Global regulator |
| *rpoS* | *△relA△rpoS* | S > D (GEN) S > D (NOR) S = D (AMP) | B (GEN) D (NOR) E (AMP) | Global regulator |
| *pspF* | *△relA△pspF* | S > D (GEN/AMP/NOR) | B (GEN/AMP/NOR) | Signaling pathway |
| *tnaA* | *△relA△tnaA* | S > D (GEN/AMP) S = D (NOR) | D (GEN/AMP) E (NOR) | Signaling pathway |
| *sucB* | *△relA△sucB* | S > D (AMP) S = D (GEN/NOR) | C (AMP) E (GEN/NOR) | Energy production |
| *ssrA* | *△relA△ssrA* | S > D (GEN /NOR) S > D (AMP) | B (GEN /NOR) D (AMP) | Trans-translation |
| *smpB* | *△relA△smpB* | S > D (AMP) S = D (GEN/NOR) | D (AMP) E (GEN/NOR) | Trans-translation |
| *recA* | *△relA△recA* | S < D (GEN/AMP) S > D (NOR) | A (GEN/AMP) B (NOR) | SOS response |
| *umuD* | *△relA△umuD* | S > D (GEN) S = D (AMP/NOR) | C (GEN) E (AMP/NOR) | SOS response |
| *uvrA* | *△relA△uvrA* | S > D (GEN/AMP) S = D (NOR) | C (GEN/AMP) E (NOR) | SOS response |
| *hipA* | *△relA△hipA* | S > D (GEN/AMP) S = D (NOR) | C (AMP) D (GEN) E (NOR) | TA module |
| *mqsR* | *△relA△mqsR* | S > D (GEN /NOR) S = D (AMP) | C (GEN /NOR) E (AMP) | TA module |
| *relE* | *△relA△relE* | S > D (GEN/AMP) S = D (AMP/NOR) | C (GEN) E (AMP/NOR) | TA module |
| *dinJ* | *△relA△dinJ* | S > D (GEN/AMP) S = D (NOR) | C (GEN/AMP) E (NOR) | TA module |
| *phoU* | ^—e^ | — | — | Global regulator |
| *oxyR* | — | — | — | Antioxidant defense |
| *glpD* | — | — | — | Energy production |
| *tisAB* | — | — | — | TA module |

^a^ The three antibiotics “GEN, AMP and NOR” in the table refer to gentamicin, ampicillin and norfloxacin, respectively. ^b^ “S” refers to persister levels of single knockout mutants. ^c^ “D” refers to persister levels of double knockout mutants of persister genes with *relA.* ^d^ “S = D” means persister levels of single knockout mutants are similar to those of double knockout mutants. ^e^ Construction of these double knockout mutants was not successful.
