# Supplementary material for: Variable Persister Gene Interactions with (p)ppGpp for Persister Formation in Escherichia coli
Source: Front Microbiol. 2017 Sep 20;8:1795. doi: 10.3389/fmicb.2017.01795 (PMC5611423; doi:10.3389/fmicb.2017.01795)
Supplement: Supplementary file 2 [file Table_2.DOCX]

Supplementary Material

Variable Persister Gene Interactions with (p)ppGpp for Persister Formation in *Escherichia coli*

Shuang Liu^1^, Nan Wu^1^, Shanshan Zhang^1^, Youhua Yuan^1^, Wenhong Zhang^1^,^*^ and

Ying Zhang^1,2,*^

*** Correspondence:**Ying Zhang
yzhang@jhsph.edu

WenHong Zhang
[zhangwenhong@fudan.edu.cn](mailto:zhangwenhong@fudan.edu.cn)

**Table S2 | Complex interactions of 11 persister genes with stringent response (p)ppGpp in the presence of three cidal antibiotics^a^ using 1:100 diluted cultures.**

| **Name** | **Double knockout mutants with *relA*** | **Persister level comparison** | **Classification** | **Persister pathways** |
| --- | --- | --- | --- | --- |
| *dnaK* | *△relA△dnaK* | S^b^ < D^c^ (GEN/AMP/NOR) | A (GEN) D (AMP/ NOR) | Global regulator |
| *clpB* | *△relA△clpB* | S = D^d^ (GEN/AMP) S > D (NOR) | E (GEN/AMP) D (NOR) | Global regulator |
| *pspF* | *△relA△pspF* | S > D (AMP/NOR) S = D(GEN) | B (AMP/NOR) E(GEN) | Signaling pathway |
| *tnaA* | *△relA△tnaA* | S > D (AMP) S = D (GEN/NOR) | D (AMP) E (GEN/NOR) | Signaling pathway |
| *sucB* | *△relA△sucB* | S = D (GEN/AMP/NOR) | E (GEN/AMP/NOR) | Energy production |
| *ssrA* | *△relA△ssrA* | S > D (GEN) S= D (AMP/NOR) | B (GEN) E (AMP/NOR) | Trans-translation |
| *smpB* | *△relA△smpB* | S > D (GEN/AMP) S = D (NOR) | B (GEN) D (AMP) E (NOR) | Trans-translation |
| *recA* | *△relA△recA* | S = D (AMP) S > D (GEN/NOR) | E (AMP) B (GEN/NOR) | SOS response |
| *hipA* | *△relA△hipA* | S = D (GEN/AMP/NOR) | E (NOR) (GEN/AMP/NOR) | TA module |
| *mqsR* | *△relA△mqsR* | S = D (GEN /NOR) S > D (AMP) | E (GEN /NOR) D (AMP) | TA module |
| *relE* | *△relA△relE* | S= D (GEN/AMP/NOR) | E (GEN/AMP/NOR) | TA module |

^a^ The three antibiotics “GEN, AMP and NOR” in the table refer to gentamicin, ampicillin and norfloxacin, respectively. ^b^ “S” refers to persister levels of single knockout mutants. ^c^ “D” refers to persister levels of double knockout mutants of persister genes with *relA.* ^d^ “S = D” means persister levels of single knockout mutants are similar to those of double knockout mutants.
